# *Lactobacillus johnsonii* BS15 improves intestinal environment against fluoride-induced memory impairment in mice—a study based on the gut–brain axis hypothesis

**DOI:** 10.7717/peerj.10125

**Published:** 2020-10-07

**Authors:** Jinge Xin, Dong Zeng, Hesong Wang, Ning Sun, Abdul Khalique, Ying Zhao, Liqian Wu, Kangcheng Pan, Bo Jing, Xueqin Ni

**Affiliations:** 1 Animal Microecology Institute, College of Veterinary Medicine, Sichuan Agricultural University, Chengdu, Sichuan, China; 2 Department of Gastroenterology, Guangdong Provincial Key Laboratory of Gastroenterology, Institute of Gastroenterology of Guangdong Province, Nanfang Hospital, Southern Medical University, Guangzhou, China

**Keywords:** Fluoride, Memory impairment, Gut-brain axis, *Lactobacillus johnsonii* BS15, Hippocampus

## Abstract

**Background:**

Excessive fluoride can lead to chronic neurodegeneration characterized by neuron and myelin loss and memory dysfunction. The gut–brain axis hypothesis suggests that gut microbiota plays a crucial role in regulating brain function. Thus, using probiotics to adjust the gut microenvironment may be a potential therapy for mental diseases.

**Methods:**

Mice in the prob group were administrated with *Lactobacillus johnsonii* BS15 for 28 days prior to and throughout a 70-day exposure to sodium fluoride. The drinking water of all groups (F and prob groups) except the control group were replaced by high-fluoride water (100 mg NaF/L) on day 28. Animals in each group were divided into two subsets: one underwent behavioral test, and the other was sacrificed for sampling. The mRNA expression level and protein content related to inflammatory reaction in the ileum and hippocampus were respectively detected by reverse transcription quantitative polymerase chain reaction (RT-qPCR) and enzyme-linked immunosorbent assay (ELISA). The mRNA expression levels of proteins related to myelin structure, apoptosis, and memory in the hippocampus and tight junction proteins in the ileum were determined by RT-qPCR and/or immunohistochemistry. Gut permeability markers (D-lactate and diamine oxidase (DAO)) in the serum were also examined by ELISA.

**Results:**

The results showed that fluoride exposure induced a lower spontaneous exploration (*P* < 0.05) in T-maze test, which indicated an impairment of memory. Spontaneous exploration of BS15-treated mice was significantly higher (*P* < 0.05) than that in F group. Fluoride reduced (*P* < 0.05) levels of myelin structural protein (proteolipid protein) and neurogenesis-associated proteins (brain-derived neurotrophic factor and cAMP/Ca^2+^ responsive element-binding protein), induced disordered inflammatory cytokines (*TNF-*α, *IFN-*γ, and *IL-6*; *P* < 0.05), increased pro-apoptotic genes (*caspase-3*; *P* < 0.05), and decreased anti-apoptotic genes (*Bcl-2*; *P* < 0.05) in the hippocampus, of which the influences were reversed by BS15. BS15 treatment exerted significant preventive effects on reversing the gut inflammation induced by excessive fluoride intake by reducing (*P* < 0.05) the levels of pro-inflammatory cytokines (tumor necrosis factor-alpha (*TNF-*α) and interferon-gamma (*IFN-*γ)) and remarkably increasing (*P* < 0.05) the level of anti-inflammatory cytokines (*IL-10*). Moreover, the serum DAO activity and D-lactate concentration significantly increased by fluoride were also reduced (*P* < 0.05) by BS15. This result indicated the profitable effect of BS15 on gut permeability.

**Conclusion:**

*L. johnsonii* BS15 intake could benefit the neuroinflammation and demyelination in the hippocampus by improving the gut environment and ameliorating fluorine-induced memory dysfunction.

## Introduction

Excessive fluoride intake has attracted increasing attention because of its widely introduced sources and adverse effects on human health. Fluoride concentration in the groundwater could range from under 1 mg/L to more than 35 mg/L (Petrone et al., 2013). In addition, fluoride concentration in drinking tea (especially Chinese brick tea) ranges from 600–2,800 mg/kg (Fung et al., 1999). The risk of high fluoride intake from food is also increasing because of the increased fluorine-containing crop protection compounds (Maienfisch & Hall, 2004). The most well-known fluoride-induced negative influences are on teeth (dental fluorosis) (Sabokseir, Golkari & Sheiham, 2016) and skeleton (skeletal fluorosis) (Littleton, 1999; Wang et al., 2019). Workers exposed to high fluoride suffer from drowsiness and impaired learning and memory (Czerwiński & Lankosz, 1978). Furthermore, epidemiological investigation from China (Wang et al., 2007), India (Sebastian & Sunitha, 2015), Mexico (Bashash et al., 2017), and Iran (Razdan et al., 2017) reported that children residing in endemic areas show deficits in learning and memory abilities. Rodents exposed to excessive fluoride also showed poor performances in memory-related behavioral tests in animal experiments (Chen et al., 2018; Liu et al., 2010). Animal experiments concerning fluoride neurotoxicity demonstrated that high fluoride exposure causes the pathological alteration of the synaptic ultrastructure of the hippocampus (Qian et al., 2013). Niu et al. (2018) suggested that fluoride could reduce neurotrophy and neuron adhesion and consequently damage the myelin in the hippocampus of mice.

Few safe and effective methods can protect the brain from fluoride neurotoxicity. The gut–brain axis, a bi-directional communication system linking the gut and brain, has provided a new insight into the treatment of brain-derived diseases (Forsythe & Kunze, 2013). Recent advances in metagenomics confirmed that the relationship between diet and gut microbiota is a critical modulator underlying neurodevelopmental and psychiatric disorders in adults (Mayer, 2011). Germ-free mice and antibiotic-treated mice have exhibited lower performance in a series of memory behavioral test than normal animals. This finding suggested that a disturbed gut microbiota is associated with memory dysfunction and brain-derived neurotrophic factor (*BDNF*) reduction (Arentsen et al., 2015; Bercik et al., 2011; Desbonnet et al., 2014). Similarly, exposure to fluoride can induce the imbalance of microbiological composition. Yasuda et al. (2017) found that fluoride exposure causes a depletion of acidogenic bacterial genera in oral community. Luo et al. (2016) reported that excessive fluoride intake induces a reduction of *Lactobacillus spp*. in the gut of broiler chickens. Moreover, previous studies demonstrated that mental diseases and cognitive functions can be effectively modulated by supplying probiotics or prebiotics to enhance the intestinal environment (Sgritta et al., 2019; Gareau et al., 2011). Therefore, we speculated that a potential link between disordered gut physiology and fluoride neurotoxicity could be utilized in preventing fluoride-induced memory dysfunction. However, little evidence has clearly demonstrated this relationship, and no probiotic has been proven effective on preventing fluoride-induced dysfunctions in the brain.

Therefore, the present study aimed to assess whether fluoride could alter gut physiology. *Lactobacillus johnsonii* BS15 was used to revert the altered intestinal physiology to further reveal whether fluoride neurotoxicity is associated with intestinal physiology and assess whether BS15 could be an effective strategy to control fluoride-induced memory dysfunction and hippocampal injury. The hippocampus is critical for bacteria–cognition link, as well as learning and memory (Stachenfeld, Botvinick & Gershman, 2017), because of its lifetime synaptic plasticity and neurogenesis (Greenberg, Ziff & Greene, 1986; Deisseroth & Tsien, 2002; Hong, Mccord & Greenberg, 2008), thus, changes in hippocampal chemistry were given more attention in this study. The expression of neuronal activity-regulated genes, such as the immediate-early gene *c-fos*, *BDNF*, and neuronal cell adhesion molecule (*NCAM*), play a critical role in synaptic plasticity (Hong, Mccord & Greenberg, 2008; Simpson & Morris, 2000). Neurotrophin- and activity-dependent gene expression is mediated by cAMP/Ca^2+^ responsive element-binding protein (*CREB*) (Mantamadiotis et al., 2002). Moreover, *BDNF*, *CREB*, *NCAM*, and *stem cell factor* (*SCF*) are essential for neurogenesis.

## Materials and Methods

### Culture and treatment with BS15

*Lactobacillus johnsonii* BS15 (CCTTCC M2013663) was isolated from homemade yogurt collected from Hongyuan Prairie, Aba Autonomous Prefecture, China. Our previous study demonstrated that BS15 can effectively prevent non-alcoholic fatty liver disease by attenuating mitochondrial lesion and inflammation in the liver, lowering intestinal permeability, and adjusting gut microbiota (Xin et al., 2014). Thus, BS15 was selected as the potential treatment strategy to improve gut flora in the present study. *L. johnsonii* BS15 was cultured anaerobically in de Man–Rogosa–Sharpe broth (Qingdao Rishui Bio-technologies Co., Ltd., Qingdao, China) at 37 °C. The amounts of bacterial cells were evaluated by heterotrophic plate count. Briefly, the cultures were centrifuged, washed, and resuspended in phosphate buffered saline (PBS; pH 7.0) for experimental use. The concentration of BS15 suspension was 1 × 10^9^ cfu/mL (daily consumption dose: 0.2 mL/mice).

### Behavioral tests

#### Novel object recognition test

The novel object recognition (NOR) test is a widely used method for the investigation of working memory alteration. The results of NOR test reflects the function of the hippocampus based on the nature propensity of mice to a novel object rather than a familiar one. The task procedure consists three phases (Antunes & Biala, 2012): habituation, familiarization, and test phase. Briefly, in the habituation phase, each mouse was allowed to freely explore the open-field arena (40 × 40 × 45 cm, l × b × h) for 1 h in the absence of objects. The mouse was then removed from the arena and placed in its home cage. During the familiarization phase, each mouse was placed in the arena to freely explore two different objects (#A + #B) for 5 min. The two objects were placed in the opposite corners of the cage. The mouse was given an intermediate retention interval of 20 min and then returned to the arena and re-exposed to object B along with a completely new object (object #C, distinguishable from object #A). Exploration ratio (F#C/(F#C + F#B) × 100, where F#C = frequency of exploring object #C, and F#B = frequency of exploring object #B) was calculated to assess memory. The objects used included a green bottle cap (#A), an orange bottle cap (#B), and a small smooth stone (#C).

#### T-maze test

An enclosed T-maze, which is an equipment with 10 cm-wide floor and 20 cm-high walls in the form of a “T”, was placed horizontally. The stems of two goal arms and a start arm were 30 cm long. A central partition in the middle of the two goal arms extended into the start arm (seven cm). Every arm had a guillotine door. The equipment and the operating steps were consistent with those of Deacon & Rawlins (2006). First, the central partition was put in the T-maze with all doors open. Then, each mouse was placed in the start area directly from its home cage and allowed to choose the left or right arm. The mouse was kept in the chosen arm by quietly sliding the door down. After 30 s, the mouse and central partition were removed, and the mouse was placed back into its holding cage. After a retention interval of 1 min, the mouse was placed back into the start area for a second trial with all doors open. Each mouse was given ten trials over 5 days and allowed to explore the maze before sated. The trial was marked as “correct” if the mouse chooses the other goal arm in consecutive trials. Each exploration should take no more than 2 min.

### Establishment of animal model and study design

Forty-eight male ICR mice (3 week-old, Dashuo Biological Institute, Chengdu, Sichuan, China) were fed with normal chow diet (Dashuo Biological Institute, Chengdu, Sichuan, China) for 1 week to acclimatize to the new environment. After the adaptation period, the mice were equally and randomly divided into three groups and administered with either 0.2 mL of PBS (control group, F group) or BS15 (prob group) every day by gavage throughout a 98-day experimental period. Animals in the F and prob groups were exposed to 100 mg/L fluoride in drinking water from the 28th day to the 98th day. The mice were housed in an animal facility with a humidity of 40–60%, a temperature of 20–22 °C and a 12-h light/dark cycle (lights off at 6:00 p.m. and on at 6:00 a.m.). We housed five or six mice per cage bedded by wood shavings and with food and water available ad libitum. The wood shavings were replaced every 2 days. Drinking water was replaced and water bottles were washed every 5 days. All animal experiments were performed according to the guidelines for the care and use of laboratory animals approved by the Institutional Animal Care and Use Committee of Sichuan Agricultural University (Approval number: SYXKchuan2019-187). Ten mice from each group were selected for behavioral test on the 98th day of the experiment. The other six mice of each group were sacrificed by cervical dislocation to collect tissues. The behavior test and sampling were carried out from 7:00 a.m. to 11:30 a.m.

Blood was sampled from the mice orbit, and the serum was separated by incubation at 4 °C for 30 min followed by centrifugation at 2,000 × g for 20 min and stored at −30 °C. Tissues from the left hippocampus and partial ileum were removed and washed with ice-cold sterilized saline and then immediately frozen in liquid nitrogen for gene expression analysis. Tissues from the right hippocampus and partial ileum were ground (pH 7.4) into 5% and 10% homogenates, respectively, with PBS and then centrifuged at 12,000× g for 5 min at 4 °C. The obtained supernatant was stored at −80 °C for further detection.

### Biochemical evaluation

The contents of corticosterone, D-lactate, and diamine oxidase (DAO) in the serum; inflammatory cytokines in the supernatant of hippocampal and ileal homogenates; and the apoptosis-regulated proteins in the supernatant of the hippocampal homogenate were measured by commercial enzyme-linked immunosorbent assay (ELISA) kit (Enzyme-linked Biotechnology Co., Ltd., Shanghai, China) specific for mice. The inflammatory cytokines included tumor necrosis factor-alpha (*TNF-*α), interferon-gamma (*IFN-*γ), interleukin-1β (*IL-1*β), *IL-6*, and *IL-10* (only detected in ileum tissue). The operation was performed strictly according to the manufacturer’s instructions.

### Real-time quantitative polymerase chain reaction analysis of gene expression

Total hippocampal RNA and ileal RNA were isolated using E.Z.N.A.® Total RNA Kit (OMEGA Bio-Tek, Doraville, GA, USA) according to the manufacturer’s instructions. The isolated RNA was assessed for the ratio of absorbances at 260 and 280 nm and by agarose gel electrophoresis for quantitative and qualitative analyses. The isolated RNA was transcribed into first-strand complementary DNA (cDNA) with PrimeScript RT reagent kit with gDNA Eraser (Thermo Scientific, Waltham, MA, USA) according to the manufacturer’s instructions. The cDNA products were stored in −80 °C for further use. qPCR was performed using CFX96 RT PCR Detection System (Bio-Rad, Hercules, CA, USA) and SYBR Premix Ex TaqTM PCR Kit (Bio-Rad, Hercules, CA, USA) to quantify the relative expression levels of neuroplasticity-related factors (*BDNF*, *CREB*, *SCF*, and *NCAM*), early gene (*c-fos*), molecular proteins related to myelin structure (myelin oligodendrocyte glycoprotein (*MOG*), proteolipid protein (*PLP*), myelin basic protein (*MBP*), and myelin-associated glycoprotein (*MAG*)), and apoptosis-related proteins (B-cell lymphoma-2 (*Bcl-2*), B-cell lymphoma-extra large (*Bcl-xl*), *Bcl-2*-associated X protein (*Bax*), *Bcl-xl*/*Bcl-2*-asociated death promoter (*Bad*), *caspase-9*, and *caspase-3*) and cytokines (*IFN-*γ, *TNF-*α, *IL-1*β, *IL-6*, and *IL-10*) in the hippocampus tissue and cytokines and tight junction (TJ) proteins (zonula occludens protein 1 (*ZO-1*), *claudin-1*, and *occludin*) in ileum tissue with 10 µL total reaction volume. The thermocycle protocol was performed as follows: 5 min at 95 °C, followed by 40 cycles of 10 s denaturation at 95 °C, and 30 s annealing/extension at optimum temperature (Table 1). A final melting curve analysis was performed to monitor the purity of the PCR product. β*-actin* was used as reference gene to normalize the relative mRNA expression levels of target genes with values presented as 2^−ΔΔCq^. The primer sequences and optimum annealing temperatures are shown in Table 1.

**Table 1:** Primer sequences for the targeted mouse genes.

	Primer sequences (5′→3′)	Annealing temp (°C)	Reference
b-actin	Forward: gctcttttccagccttcctt Reverse: gatgtcaacgtcacactt	60	Niu et al. (2018)
BDNF	Forward: gcgcccatgaaagaagtaaa Reverse: tcgtcagacctctcgaacct	60	Niu et al. (2018)
NCAM	Forward: gggaactccatcaaggtgaa Reverse: ttgagcatgacgtggacact	60	Niu et al. (2018)
SCF	Forward: ccttatgaagaagacacaaacttgg Reverse: ccatcccggcgacatagttgaggg	60	Niu et al. (2018)
CREB	Forward: ccagttgcaaacatcagtgg Reverse: ttgtgggcatgaagcagtag	60	Niu et al. (2018)
MOG	Forward: aaaacaccctgtggtgaagg Reverse: atcctggttggcagaatcac	60	Niu et al. (2018)
MAG	Forward: gttcctcagctcctcattgc Reverse: ttggggatgtctcctgattc	60	Niu et al. (2018)
MBP	Forward: atccaagtacctggccacag Reverse: cctgtcaccgctaaagaagc	60	Niu et al. (2018)
PLP	Forward: caggctcctgctagaaatgg Reverse: ggtcttcaggagatgcttgc	60	Niu et al. (2018)
c-fos^a^	Forward: cagagcgggaatggtgaaga Reverse: ctgtctccgcttggagtgta	59.5	
TNF-α	Forward: acggcatggatctcaaagac Reverse: agatagcaaatcggctgacg	60	Xin et al. (2014)
IL-1β	Forward: atgaaagacggcaccccac Reverse: gcttgtgctctgcttgtgag	60	Ren et al. (2013)
IL-6	Forward: tgcaagagacttccatccagt Reverse: gtgaagtagggaaggccg	60	Ren et al. (2013)
IL-10	Forward: ggttgccaagccttatcgga Reverse: acctgctccactgccttgct	60	Jaini et al. (2010)
IFN-γ	Forward: tcaagtggcatagatgtggaagaa Reverse:tggctctgcaggattttcatg	60	Jaini et al. (2010)
ZO-1	Forward: gatccctgtaagtcacccaga Reverse: ctccctgcttgcactcctatc	60	Zhong et al. (2010)
Claudin-1	Forward: ggggacaacatcgtgaccg Reverse: aggagtcgaagactttgcact	60	Zhong et al. (2010)
Occludin	Forward: ttgaaagtccacctccttacaga Reverse: ccggataaaaagagtacgctgg	60	Zhong et al. (2010)

**Note:**

a The primer sequences of c-fos is designed by National Center for Biotechnology Information (NCBI) and the referenced gene ID is 14281.

### Immunohistochemistry

A subset of mice in each group was sacrificed, and their brain was removed, fixed in 4% paraformaldehyde solution, and stored in 4 °C for immunohistochemical assay. The brain tissues were embedded by paraffin and cut by microtome. Slices were submerged in citrate antigen retrieval solution and heated on medium until boiling using a microwave (model: P70D20TL-P4; Galanz, Guangdong, China). The fire was ceased, and the tissues were kept warm for 8 min. Then, the tissues were heated at medium–low heat for 7 min. After free cooling, the slices were placed into PBS (pH 7.4) and shaken for 5 min for decoloration, which was repeated three times. Then, the sections were incubated in 3% oxydol for 25 min at room temperature and away from light for blocking endogenous peroxidase. The slices were washed three times in PBS by shaking for 5 min, then sealed for 30 min by 3% bull serum albumin, and incubated with monoclonal rabbit anti-*BDNF* (1:400) or polyclonal rabbit anti-*CREB* (1:500) at 4 °C overnight. Species-specific biotinylated anti-rabbit immunoglobulin (horseradish peroxidase-labeled) was used for immuno-detection. Following the second antibody incubation, 3,3′-diaminobenzidine staining kit was used to complete the reaction according to the manufacturer’s instructions. Hematoxylin stain was performed to re-stain the nucleus. *BDNF* and *CREB* were quantified by calculating their integral optical density (IOD) in the object region (ImageJ; National Institutes of Health, Bethesda, MD, USA). The average optical density (IOD/object region areas) was calculated, and the results were presented as levels of expression.

## Statistics

Results were expressed as mean ± standard deviation. One-way ANOVA was performed between different groups with IBM SPSS Statistics 25 (IBM Corporation). Differences of *P* < 0.05 were considered statistically significant. The figures were plotted using GraphPad Prism version 7.04 (San Diego, CA, USA).

## Results

### Behavioral results

**Figure 1:**
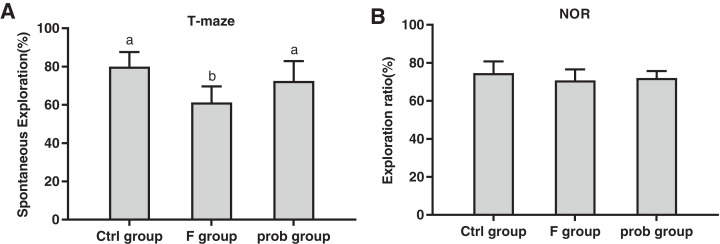
Results of behavioral tests. (A) T-Maze and (B) NOR preference tests. Data are presented as mean ± standard deviation (T-maze, *n* = 8; NOR, *n* = 10). Bars with different letters (a, b, and c) indicate significant difference on the basis of Duncan’s multiple range test (*P* < 0.05).

Figure 1A shows that the F group showed a lower spontaneous exploration (control vs. F, *P* < 0.01; F vs. prob, *P* = 0.019) than the other two groups, but no difference (*P* > 0.05) was found between the control and prob groups (*P* = 0.104). The exploration ratio of the three groups were not significantly different (control vs. F, *P* = 0.125; control vs. prob, *P* = 0.285; F vs. prob, *P* = 0.626; Fig. 1B).

### mRNA and protein expressions of *BDNF* in the hippocampus

**Figure 2:**
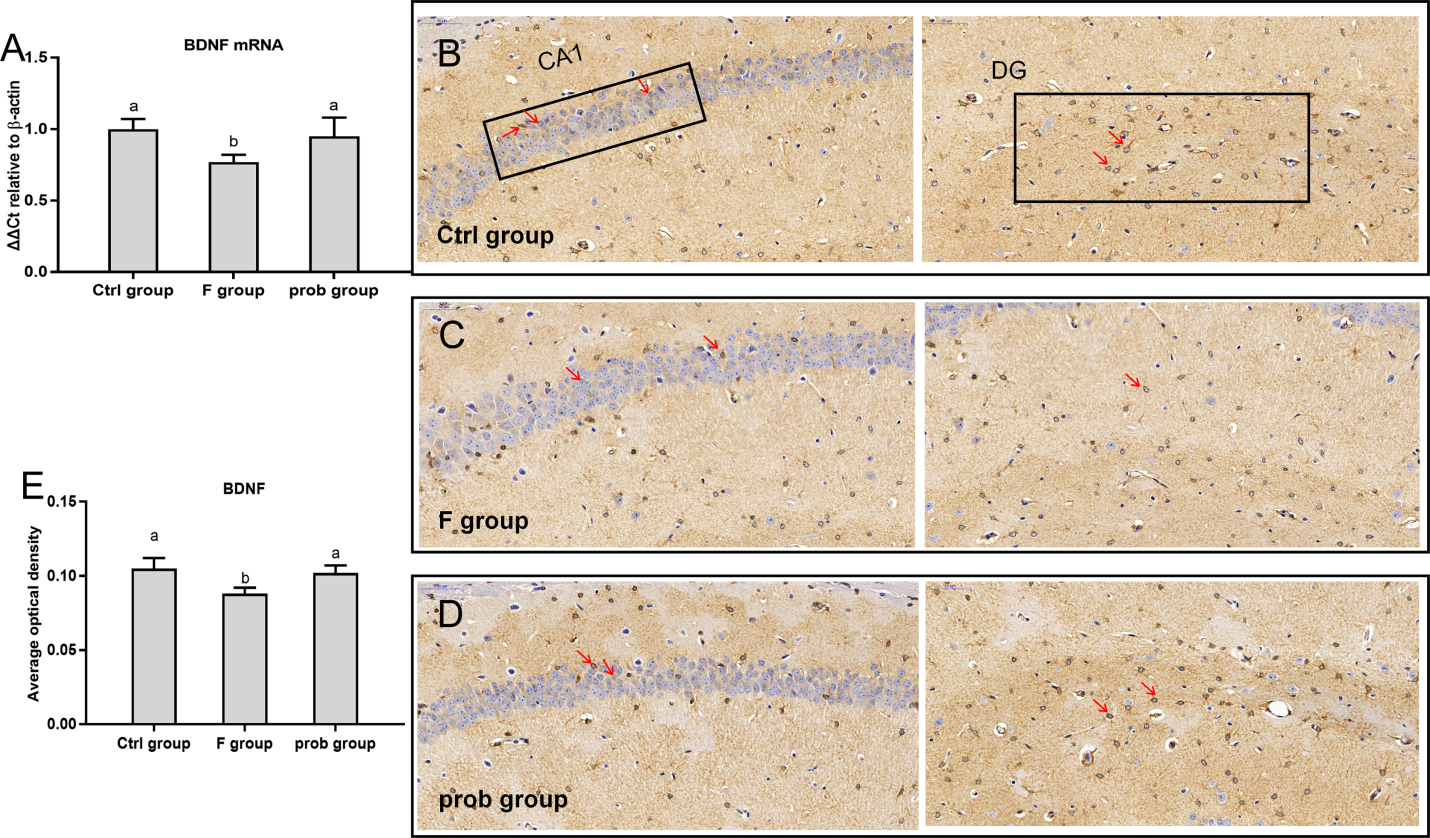
*BDNF* expression in the hippocampus of mice. (A) mRNA expression level of *BDNF* in the hippocampus(*n* = 4–5). (B–D) The images labeled B, C, and D present results of immunohistochemistry in control, F, and prob groups, respectively (*n* = 3). (E) Results of *BDNF* immunohistochemistry were quantified, and the results are presented in the figure 2E. The *BDNF*-positive cells are brown. Data are presented as mean ± standard deviation. Bars with different lowercase letters (a, b, and c) indicate significant difference on the basis of Duncan’s multiple range test (*P* < 0.05). DG: dentategyrus.

Figure 2A shows that the F group presented a significant decrease in *BDNF* mRNA level than the other two groups (control vs. F, *P* < 0.01; F vs. prob, *P* = 0.014), whereas the control and prob groups had no difference (*P* = 0.414). As shown in Fig. 2B–2E, the *BDNF* protein level was decreased in the F group compared with the control (*P* < 0.01) and prob groups (*P* = 0.018). No difference was observed between the control and prob groups (*P* = 0.513).

### mRNA and protein expressions of *CREB* in the hippocampus

**Figure 3:**
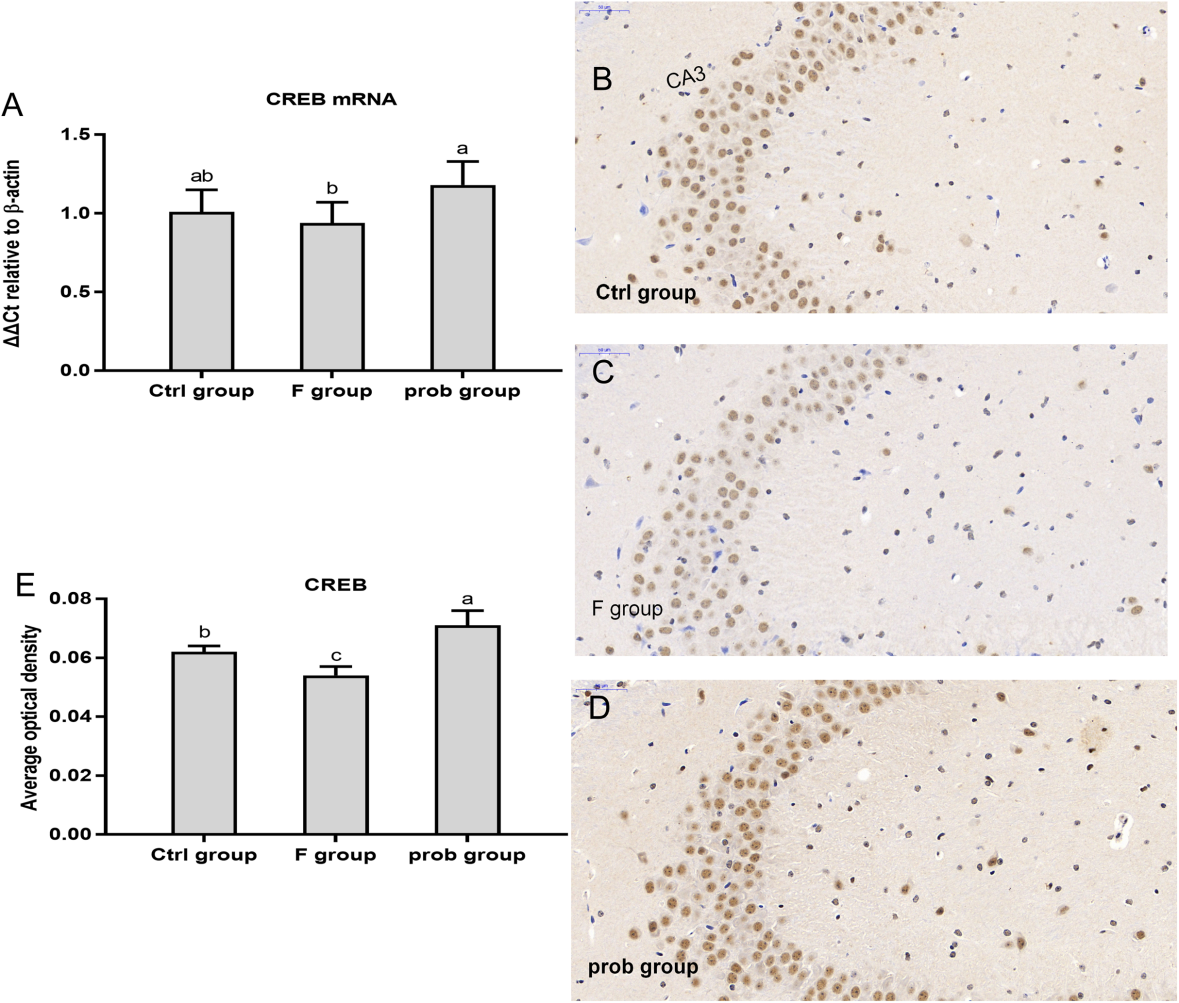
*CREB* expression in the hippocampus of mice. (A) mRNA expression level of *CREB* in the hippocampus(*n* = 4–5). (B–D) The images labeled B, C, and D present results of immunohistochemistry in control, F, and prob groups, respectively (*n* = 3). (E) Results of *CREB* immunohistochemistry were quantified, and the results are presented in the figure 2E. The *CREB*-positive cells are brown. Data are presented as mean ± standard deviation. Bars with different lowercase letters (a, b, and c) indicate significant difference on the basis of Duncan’s multiple range test (*P* < 0.05).

As shown in Fig. 3A, *CREB* mRNA level was slightly reduced in the F group (*P* = 0.484) and increased in the prob group (*P* = 0.065) compared with the control group. The *CREB* mRNA level in the prob group was significantly (*P* = 0.026) higher than that in the F group. Figures 3B–3E shows that the F group presented a significant decrease in *CREB* protein level than the other two groups (control vs. F, *P* = 0.048; F vs. prob, *P* < 0.01). *CREB* protein level in the prob group was remarkably higher than that in the control group (*P* = 0.032).

### mRNA expressions of *NCAM*, *SCF*, and *c-fos* in the hippocampus

**Figure 4:**
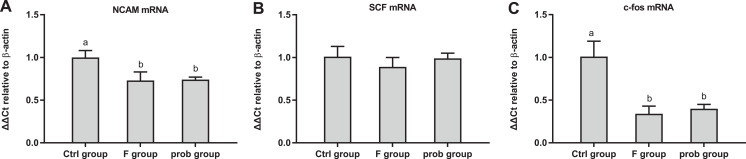
mRNA expressions of *NCAM*, *SCF*, and *c-fos* in the hippocampus. Relative expression levels of (A) *NCAM*, (B) *SCF*, and (C) *c-fos*. Data are presented as mean ± standard deviation (*n* = 4–5). Bars with different lowercase letters (a, b, and c) indicate significant difference on the basis of Duncan’s multiple range test (*P* < 0.05).

As shown in Fig. 4A, the control group had a higher *NCAM* mRNA level than the other two groups (control vs. F, *P* = 0.001; control vs. prob, *P* = 0.001), but no difference was found between the F and prob groups (*P* = 0.783). Figure 4B shows that the *SCF* mRNA level of the three groups were not significantly different (control vs. F, *P* = 0.105; control vs. prob, *P* = 0.842; F vs. prob, *P* = 0.181). As shown in Fig. 4C, the control group presented a higher mRNA expression of c-fos than the other two groups (control vs. F, *P* < 0.01; control vs. prob, *P* < 0.01), but the expression of c-fos between the other two groups were not significantly different (F vs. prob, *P* = 0.449).

### Hippocampal inflammation

**Figure 5:**
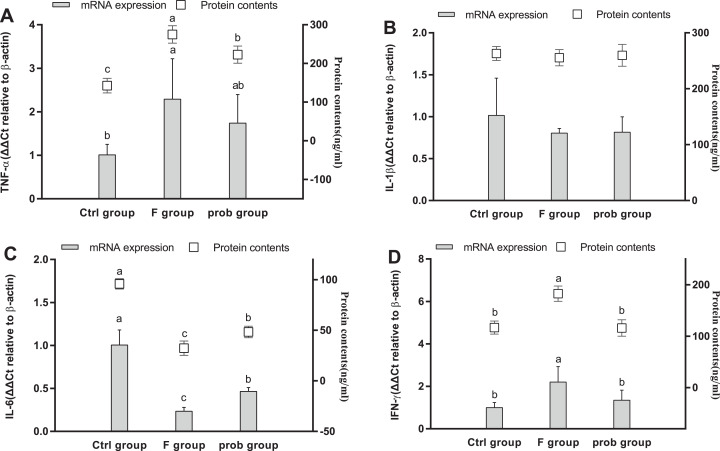
mRNA and protein expression levels of inflammatory cytokines in the hippocampus. Relative expression levels of (A) *TNF-*α, (B) *IL-*β, (C) *IL-6*, and (D) *IFN-*γ. Data are presented as mean ± standard deviation (*n* = 4–6). Bars with different lowercase letters (a, b, and c) indicate significant difference on the basis of Duncan’s multiple range test (*P* < 0.05).

Figures 5A and 5D show that the *TNF-*α (mRNA level: control vs. F, *P* = 0.013; F vs. prob, *P* = 0.252; protein level: control vs. F, *P* < 0.001; F vs. prob, *P* = 0.001) and *IFN-*γ (mRNA level: control vs. F, *P* = 0.007; F vs. prob, *P* = 0.037; protein level: control vs. F, *P* < 0.001; F vs. prob, *P* < 0.001) in the F group were significantly or slightly higher than those in the other two groups in mRNA and protein levels. The *TNF-*α of the prob group was significantly increased in the protein level (*P* < 0.001) but not in the mRNA level (*P* = 0.115) compared with the control group (Fig. 5A). No difference in *IFN-*γ (Fig. 5D) was observed between the control and prob groups in the mRNA (*P* = 0.381) and protein levels (*P* = 0.931). Figure 5C reveals that the F group had a remarkably lesser *IL-6* than the other two groups in mRNA (control vs. F, *P* < 0.001; F vs. prob, *P* = 0.011) and protein levels (control vs. F, *P* < 0.001; F vs. prob, *P* < 0.001). As shown in Fig. 5C, the prob group presented a significantly lesser *IL-6* than the control group in mRNA (*P* < 0.001) and protein levels (*P* < 0.001). The three groups had no remarkable change in *IL-1*β in the mRNA and protein levels (mRNA level: control vs. F, *P* = 0.089; control vs. prob, *P* = 0.097; F vs. prob, *P* = 0.962; protein level: control vs. F, *P* = 0.420; control vs. prob, *P* = 0.733; F vs. prob, *P* = 0.636). *IL-10* did not reach the detection threshold (Fig. 5B).

### Hippocampal myelin and apoptosis-related proteins

**Figure 6:**
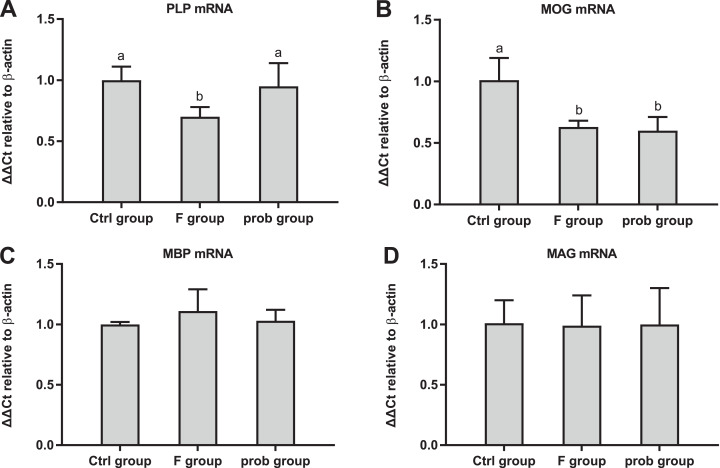
mRNA expression levels of myelin-associated proteins in the hippocampus. Relative expression levels of (A) *PLP*, (B) *MOG*, (C) *MBP*, and (D) *MAG*. Data are presented as mean ± standard deviation (*n* = 3–5). Bars with different lowercase letters (a, b, and c) indicate significant difference on the basis of Duncan’s multiple range test (*P* < 0.05).

**Figure 7:**
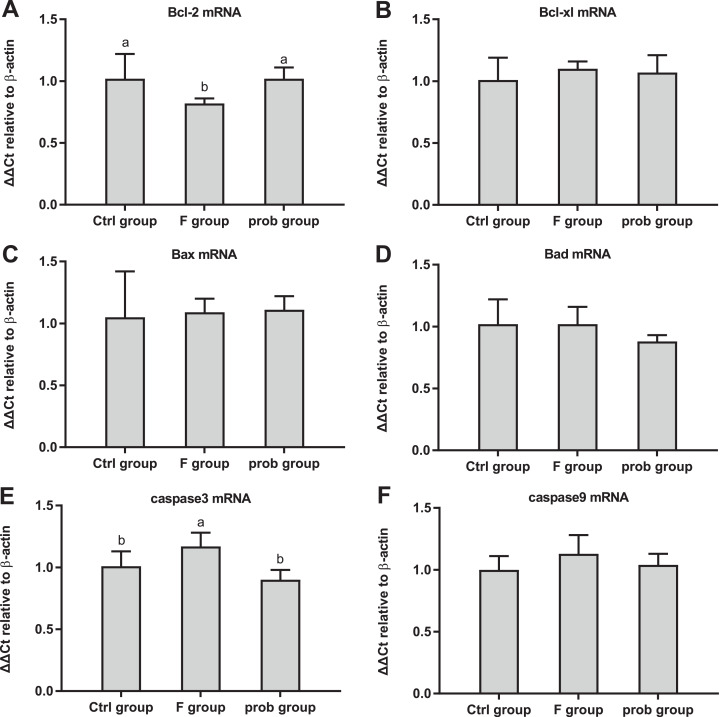
mRNA expressions levels of apoptosis-associated proteins in the hippocampus of mice. Relative expression levels of (A) *Bcl-2*, (B) *Bcl-xl*, (C) *Bax*, (D) *Bad*, (E) *caspase-3*, and (F) *caspase-9*. Bars with different lowercase letters (a, b, and c) are significantly different (*P* < 0.05) on the basis of one-way ANOVA. Each bar represents mean ± standard deviation (*n* = 4–6).

Figure 6A shows the clear decreasing trend in the mRNA level of *PLP* in the F group compared with the other two groups (control vs. F, *P* < 0.013; F vs. prob, *P* = 0.0039). A difference (control vs.prob, *P* = 0.568) in the mRNA level of *PLP* between the control and prob groups was not detected. Figure 6B shows that the control group had a higher *MOG* mRNA level than the F and prob group (control vs. F, *P* = 0.005; control vs. prob, *P* = 0.002), whereas the *MOG* mRNA level of the F group was not different (*P* = 0.778) from that of the prob group. Differences in the mRNA levels of *MBP* (control vs. F, *P* = 0.277; control vs.prob, *P* = 0.706; F vs. prob, *P* = 0.415; Fig. 6C) and *MAG* (control vs. F, *P* = 0.904; control vs. prob, *P* = 0.919; F vs. prob, *P* = 0.970; Fig. 6D) were not observed among the three groups. Figures 7A and 7E present a remarkable (control vs. F, *P* < 0.026; F vs. prob, *P* = 0.038) decrease in *Bcl-2* mRNA level and a significant (control vs. F, *P* < 0.033; F vs. prob, *P* = 0.001) increase in *caspase-3* mRNA level in F group compared with the other two groups, and the *Bcl-2* (*P* = 0.948) and *caspase-3* mRNA levels (*P* = 0.127) of the control and prob groups were not significantly different. Figures 7B, 7C, 7D, and 7F reveal no significant differences (*P* > 0.05) among the three group in the mRNA levels of *Bcl-xl* (control vs. F, *P* = 0.290; control vs. prob, *P* = 0.463; F vs. prob, *P* = 0.735), *Bax* (control vs. F, *P* = 0.784; control vs. prob, *P* = 0.681; F vs. prob, *P* = 0.891), *Bad* (control vs. F, *P* = 0.954; control vs. prob, *P* = 0.137; F vs. prob, *P* = 0.124), and *caspase-9* (control vs. F, *P* = 0.128; control vs. prob, *P* = 0.684; F vs. prob, *P* = 0.197).

### Inflammatory factors in the ileum

**Figure 8:**
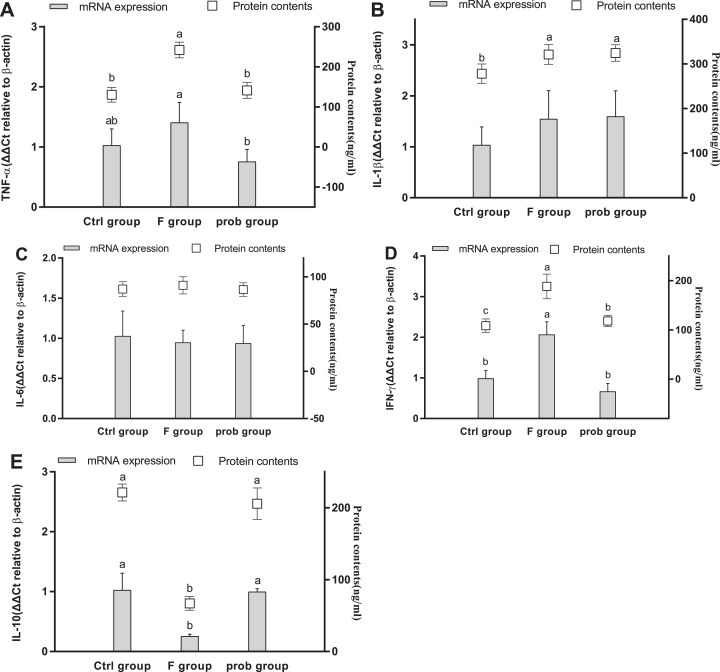
mRNA and protein expression levels of inflammatory cytokines in the ileum. Relative expression levels of (A) *TNF-*α, (B) *IL-*β, (C) *IL-6*, (D) *IFN-*γ, and (E) *IL-10*. Data are presented as mean ± standard deviation (*n* = 4–6). Bars with different lowercase letters (a, b, and c) are significantly different on the basis of Duncan’s multiple range test (*P* < 0.05).

As shown in Fig. 8A, the F group exhibited a slight increase in *TNF-*α mRNA level (*P* = 0.079) and a significantly enhanced *TNF-*α protein level (*P* < 0.001) compared with the control group. *TNF-*α in the mRNA (*P* = 0.008) and protein levels (*P* < 0.001) were significantly reduced in the prob group compared with the F group. Differences in the mRNA (*P* = 0.197) and protein levels (*P* = 0.348) of *TNF-*α were not observed between the control and prob groups. The mRNA (*P* = 0.888) and protein levels (*P* = 0.781) of *IL-1*β (Fig. 8B) between the F and prob groups were not significantly different. *IL-1*β (Fig. 8B) in the control group showed a slight decrease in mRNA level (control vs. F, *P* = 0.132; control vs. prob, *P* = 0.103) and a remarkable (control vs. F, *P* = 0.003; control vs. prob, *P* = 0.002) decline in protein level compared with those in the other two groups (Fig. 8B). No difference was observed in *IL-6* (Fig. 8C) in mRNA (control vs. F, *P* = 0.635; control vs. prob, *P* = 0.615; F vs. prob, *P* = 0.975) and protein levels (control vs. F, *P* = 0.407; control vs. prob, *P* = 0.908; F vs. prob, *P* = 0.347) among the three groups. The F group exhibited a significantly increased *IFN-*γ (mRNA level: control vs. F, *P* < 0.001; F vs. prob, *P* < 0.001; protein level: control vs. F, *P* < 0.001; F vs. prob, *P* < 0.001; Fig. 8D) and a sharply decreased *IL-10* (mRNA level: control vs. F, *P* = 0.01; F vs. prob, *P* = 0.02; protein level: control vs. F, *P* < 0.001; F vs. prob, *P* < 0.001; Fig. 8E) compared with the other two groups in mRNA and protein levels. These differences (*IFN-*γ mRNA level: control vs. prob, *P* = 0.062; *IL-10* mRNA level: control vs. prob, *P* = 0.838; *IL-10* protein level: control vs. prob, *P* = 0.535) were not observed in the other two groups except for *IFN-*γ protein level (control vs. prob, *P* = 0.005).

### Intestinal permeability

**Figure 9:**
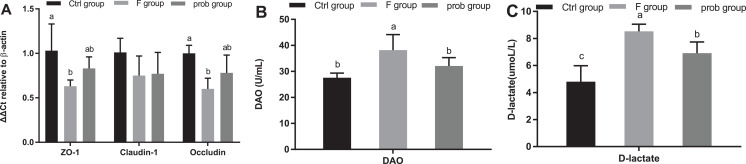
Results of intestinal permeability. (A) mRNA expression levels of *ZO-1*, *claudin-1*, and *occludin* in the ileum, (B) Serum DAO activity, and (C) Serum D-lactate concentration. Data are presented as mean ± standard deviation (*n* = 4–6). Bars with different lowercase letters (a, b, and c) indicate significant difference on the basis of Duncan’s multiple range test (*P* < 0.05).

As shown in Fig. 9A, the control group reported a slightly higher *claudin-1* than the other two groups (control vs. F, *P* = 0.111; control vs. prob, *P* = 0.129). The mRNA expression of *claudin-1* between the F and prob groups had no difference (F vs. prob, *P* = 0.855). Figure 9A also shows that *ZO-1* and *occludin* in the control group were remarkably higher than those in the F group (*P* = 0.008, *P* = 0.004) and slightly (*P* = 0.121, *P* = 0.061) higher than the prob group. Figure 9A shows that the prob group presented a slightly more *ZO-1* (*P* = 0.125) and *occludin* (*P* = 0.114) than the F group. Figures 9B and 9C show that the F group exhibited a significant increase in serum DAO activity (control vs. F, *P* = 0.002; F vs. prob, *P* = 0.048) and D-lactate concentration (control vs. F, *P* < 0.001; F vs. prob, *P* = 0.011) compared with the other two groups. No significant (*P* = 0.128) difference in the DAO activity between the control and prob groups was observed. The D-lactate concentration in the prob group was significantly (*P* = 0.001) higher than that in the control group.

## Discussion

Although a large number of studies have found fluoride-induced brain lesions, few studies focused on the link between the gut changes and neurotoxicity induced by fluoride. In view of the gut–brain axis, changes in the intestinal microenvironment, including gut microbiota, inflammatory cytokines, and hormones, can influence brain chemistry and behaviors. The current study further explored fluoride-induced brain lesion and its link with the gut. We found that the mice exposed to 100 mg/L sodium fluoride for 10 weeks had hippocampal lesions, which caused memory impairment as indicated by their lower performance in the T-maze test and the reduced *PLP* mRNA level. Moreover, high fluoride exposure caused intestinal inflammation and increased the intestinal permeability as indicated by the increased inflammatory cytokines, serum DAO activity, and D-lactate content and reduced mRNA levels of TJ protein. BS15, a probiotic capable of improving the gut microbiome, reversed the gut changes and alleviated the brain lesion and memory impairment induced by fluoride. The current study confirmed our hypothesis that gut changes may play a key role in memory dysfunction during high fluoride exposure, and BS15 administration is a potential method of preventing these fluoride-induced damages on memory ability.

Although the memory abilities of the three groups were not remarkably different as shown in the NOR test, the fluoride-infected mice in the F group showed memory impairment as indicated by their lower spontaneous exploration in the T-maze test compared with the other groups. The poor performances of the fluoride-infected mice in various memory-related behavioral tests were also reported in previous studies (Liu et al., 2010; Chen et al., 2018). In our study, BS15 substantially reversed the performance of mice in the T-maze and thus has a beneficial effect on the memory ability of fluoride-infected mice. Neuronal activation was also decreased as indicated by the lower mRNA level of *c-fos* in the hippocampus of fluoride-infected mice. Fleischmann et al. (2003) demonstrated that mice lacking *c-fos* exhibit remarkable memory deficits; hence, *c-fos* gene has a critical role in memory. The hippocampus is a unique area of the brain that is able of neuroplasticity (Galea et al., 2013). Neuroplasticity usually increases under the conditions that increase memory abilities, and its ablation often induces lesions that affect memory (Deng, Aimone & Gage, 2010; Leuner & Gould, 2010). These findings suggested that the hippocampus play a critical role in memory and synaptic plasticity (Yirmiya & Goshen, 2011). Neurotrophic factors, especially the BDNF, are important in neurogenesis, and their production are involved in almost every aspect of neural and behavioral plasticity (Lu, Christian & Lu, 2008; Li et al., 2008), especially for hippocampal-dependent memory (Heldt et al., 2007). An increasing body of data indicated that probiotic consumption can regulate anxiety and memory functions via changes in the expression of key components, such as *BDNF*, *CREB*, and N-methyl-d-aspartate receptors (Clarke et al., 2013; Wall et al., 2010). The underlying mechanism is that probiotics can synthesize and recognize an array of neurochemicals, including neurotransmitters, secondary bile acids, neuroactive short chain fatty acids (SCFAs), and other biologically active small molecules. For example, Kumar et al. (2017) reported that *L. johnsonii* could increase the concentrations of acetate and butyrate in feces. Butyrate, an SCFA, can decrease *BDNF* methylation and consequently cause an overexpression of *BDNF* by decreasing ten-eleven translocation methylcytosine dioxygenase 1, which is the enzyme responsible for catalyzing the conversion of DNA methylation to hydroxymethylation (Wei et al., 2015). In the present study, the expression of *BDNF* in the hippocampus was reduced by fluoride. This finding is consistent with the result found by Niu et al. (2018) *CREB* is the transcriptional regulator of *BDNF*, and similar genomic network analysis reported that *CREB* is the center of Alzheimer’s disease’s pathology (Jeong et al., 2001). The mRNA and protein levels of *BDNF* and *CREB* in the BS15-treated mice were substantially increased compared with the fluoride-infected mice, and the level of *CREB* was even slightly higher than the control group. Similarly, Kadry & Megeed (2018) found that *Lactobacillus* could effectively inhibit the reduction of BDNF induced by cadmium chloride in the hippocampus of mice. Moreover, Corpuz et al. (2018) reported that *Lactobacillus paracasei* K71 prevents age-related cognitive decline in senescence-accelerated mouse prone 8 by increasing the protein expression of *BDNF* and the phosphorylation of *CREB* in the hippocampus. Neuroplasticity also needs the regulation of *NCAM* (Seidenfaden, Krauter & Hildebrandt, 2006) and the stimulation of SCF (Jin et al., 2002). In line with the results of Niu et al. (2018), fluoride significantly reduced the mRNA level of *NCAM*.

Many evidence indicated that neuroinflammation may be one of the pathogenesis underlying cognitive changes and the development of many neurodegenerative diseases (Frank-Cannon et al., 2009). The hippocampus is vulnerable to the insults by inflammatory cytokines because of its high expression of cytokine receptors (Das & Basu, 2008). Few in vivo studies focused on the neuroinflammation caused by fluorosis. Excessive exposure to fluoride triggered neuroinflammation as indicated by the increase in the *TNF-*α and *IFN-*γ in the hippocampus of the F group. The result on *TNF-*α was in agreement with the finding of Yan et al. (2016), who found that *TNF-*α immunoreactivity is increased in the hippocampus of rat exposed to 120 ppm fluoride for 10 weeks. However, by contrast to the results shown by Yan et al. (2016), the present study reported a reduced *IL-6* in the hippocampus of mice exposed to 100 mg/L sodium fluoride for 10 weeks. Differences in drug doses, delivery cycles, and breeds of rodents may be associated with the contradictory results. A previous research confirmed that *IL-6*-deficient mice present a weakened neuroprotection of the hippocampus (Jean Harry, Bruccoleri & D’Hellencourt Lefebvre, 2003). Funk et al. (2011) indicated the potential role of *IL-6* in modulating *TNF-*α-mediated neurotoxicity. Intestinal microbiome and some probiotics can influence health status and disease risk by activating immune response against dangerous stimuli and activating regulatory mechanisms to avoid uncontrolled inflammation. Intestinal permeability and potentially beneficial metabolites may be the underlying mechanisms of the anti-inflammation. Intestinal microbiome can ferment dietary fiber and starch in the large intestines and produce SCFAs (Chen, Faller & Spanjaard, 2003). The effect of butyrate and other SCFAs on preventing inflammation in colon diseases and different neural inflammation models in cell cultures have been demonstrated (Huuskonen et al., 2004). In the present study, BS15 administration had profitable effect on balancing the inflammatory cytokines in fluoride-infected mice.

The mRNA expression levels of myelin- and apoptosis-related proteins were also detected to further investigate the effect of BS15 on the hippocampal impairment. Myelin sheaths enwrap the nerve fiber to guarantee interneuronal transmission efficiency (Nguyen et al., 2009). Myelin is consisted of *PLP* (a transmembrane protein), *MBP* (a peripheral membrane protein), the outermost *MOG*, and the innermost *MAG* (Niu et al., 2018). The remarkably reduced mRNA expression levels of PLP and *MOG* in the F group suggested that myelin lesion occurred in the hippocampus. The changes in *PLP* induced by fluoride were consistent with the findings of Niu et al. (2018). The reduced tendency of *PLP* was inhibited by BS15; hence, BS15 may have a protective effect on the myelin. A previous study demonstrated that mice present increased positive apoptotic neurons following 10 weeks of exposure to 120 ppm fluoride in drinking water (Yan et al., 2016). In the present study, the reduced *Bcl-2* (anti-apoptosis protein) and increased *caspase-3* (pro-apoptosis protein) in fluoride-infected mice created conditions for apoptotic neurons, and these changes were remarkably reversed by BS15 treatment.

Intestinal leakage can facilitate the translocation of bacterial composition, such as microorganisms and their products (Carvalho, Berk & Maes, 2016), and is considered a key factor in mental disease (Braniste et al., 2014; Zhan et al., 2016; Emery et al., 2017). Inflammation can enhance epithelial permeability (Xue et al., 2014; Schulzke et al., 2009). Inflammatory cytokines, such as *IL-1*β, *TNF-*α, and *IFN-*γ, can increase gut permeability (Ma et al., 2004; Schulzke et al., 2009; Weber et al., 2010). *IL-10*, an anti-inflammatory cytokine, plays a critical role in the homeostasis of the gut, which was illustrated by the finding that spontaneous colitis occurs in *IL-10*^−/−^ mice (Gomes-Santos et al., 2012). The current study found that excessive fluoride intake resulted in intestinal inflammation by increasing pro-inflammatory cytokines (*TNF-*α, *IL-1*β, and *IFN-*γ) and reducing anti-inflammatory cytokine (*IL-10*). Treatment with BS15 efficiently lowered the inflammatory reaction caused by fluoride. TJ proteins act as a barrier that mediates the cell-to-cell adhesion and prevents molecules from crossing through the epithelial sheet between adjacent cells into systemic circulation (Piche, 2014). The mRNA level of two TJ proteins, namely, *ZO-1* and *occludin*, in the ileum of the fluoride-infected mice were also remarkably reduced with gut inflammation enhancement and therefore led to higher levels of DAO activity and D-lactate content in the serum. The tissue of the small intestine contains the highest DAO activity, and serum DAO is derived primarily from the small intestines in many mammalian species (Luk, Bayless & Baylin, 1980). Moreover, mammalian species cannot produce D-lactate, and the main source of D-lactate is from the commensal bacteria in the gastrointestinal tract (Sun et al., 2001). The metabolism of serum D-lactate is very slow. The increases in serum DAO activity and D-lactate content occurred when the intestinal mucosal integrity was damaged and served as useful plasma markers of mucosal integrity (Ewaschuk, Naylor & Zello, 2005; Luk, Bayless & Baylin, 1980). In this study, we found that BS15 effectively improved intestinal permeability as shown by the remarkably lower serum DAO activity and D-lactate concentration in the prob group compared with the F group. The result may be explained in part by the slightly increased TJ proteins in the prob group. Apoptosis is another possible reason that may have caused barrier dysfunction (Schulzke et al., 2009). These results suggested that fluoride could cause intestinal inflammation and damage mucosal integrity, which results in enhanced intestinal permeability, and BS15 administration could alleviate these pathological changes.

## Conclusions

The study deepened our understanding of the link between fluoride neurotoxicity on memory function and gut microenvironment. BS15 exerted beneficial effects against excessive fluoride intake-induced memory impairment, related neural inflammation, and demyelination by improving intestinal inflammation and integrity and increasing apoptosis markers in the hippocampus of mice.

## Supplemental Information

10.7717/peerj.10125/supp-1 Supplemental Information 1 Raw measurements. Indexes of intestinal tract, hippocampus and behavioral test are in different sheets respectively.

## Additional Information and Declarations

### Data Availability

The following information was supplied regarding data availability: The raw measurements are available as a Supplemental File.
